# UBR-5, a Conserved HECT-Type E3 Ubiquitin Ligase, Negatively Regulates Notch-Type Signaling in *Caenorhabditis elegans*

**DOI:** 10.1534/g3.116.027805

**Published:** 2016-05-13

**Authors:** Komal Safdar, Anniya Gu, Xia Xu, Vinci Au, Jon Taylor, Stephane Flibotte, Donald G. Moerman, Eleanor M. Maine

**Affiliations:** * Department of Biology, Syracuse University, New York 13244; † Department of Zoology, University of British Columbia, Vancouver, British Columbia V6T 1Z4, Canada

**Keywords:** Notch, germ cell, GLP-1, LIN-12, HECT domain

## Abstract

Notch-type signaling mediates cell−cell interactions important for animal development. In humans, reduced or inappropriate Notch signaling activity is associated with various developmental defects and disease states, including cancers. *Caenorhabditis elegans* expresses two Notch-type receptors, GLP-1 and LIN-12. GLP-1 mediates several cell-signaling events in the embryo and promotes germline proliferation in the developing and adult gonad. LIN-12 acts redundantly with GLP-1 in certain inductive events in the embryo and mediates several cell−cell interactions during larval development. Recovery of genetic suppressors and enhancers of *glp-1* or *lin-12* loss- or gain-of-function mutations has identified numerous regulators of GLP-1 and LIN-12 signaling activity. Here, we report the molecular identification of *sog-1*, a gene identified in screens for recessive suppressors of conditional *glp-1* loss-of-function mutations. The *sog-1* gene encodes UBR-5, the sole *C. elegans* member of the UBR5/Hyd family of HECT-type E3 ubiquitin ligases. Molecular and genetic analyses indicate that the loss of *ubr-5* function suppresses defects caused by reduced signaling via GLP-1 or LIN-12. In contrast, *ubr-5* mutations do not suppress embryonic or larval lethality associated with mutations in a downstream transcription factor, LAG-1. In the gonad, *ubr-5* acts in the receiving cells (germ cells) to limit GLP-1 signaling activity. SEL-10 is the F-box component of SCF^SEL-10^ E3 ubiquitin–ligase complex that promotes turnover of Notch intracellular domain. UBR-5 acts redundantly with SEL-10 to limit Notch signaling in certain tissues. We hypothesize that UBR-5 activity limits Notch-type signaling by promoting turnover of receptor or limiting its interaction with pathway components.

The highly conserved Notch-type signaling process mediates inductive cell interactions during animal development (see reviews by Greenwald and Kovall 2013; Koch *et al.* 2013; Suresh and Irvine 2015; Yamamoto *et al.* 2014). Notch signaling is active in many different tissues in any given species, and defective Notch signaling is associated with many human disease conditions, including developmental syndromes and certain cancers (see reviews by Louvi and Artavanis-Tsakonas 2012; Ntziachristos *et al.* 2014; Penton *et al.* 2012; Suresh and Irvine 2015). Notch-type signaling is unusual compared with other developmentally important signaling mechanisms in that it is limited to adjacent cells, involves cleavage of the receptor to release a transcription factor, and acts in a relatively diverse set of developmental and physiological contexts. During canonical Notch signaling, summarized in Figure 1A, the DSL (Delta, Serrate, LAG-2) -type ligand on the signaling cell binds membrane-associated Notch-type receptor on the receiving cell, and this interaction triggers proteolytic cleavage of the receptor. Sequential cleavage events, accomplished by ADAM protease (the S2 cleavage) and γ-secretase (the S3 cleavage), release the Notch intracellular domain (NICD) for transport to the nucleus where it interacts with a CSL (CBF1/Su(H)/LAG-1)-type DNA binding protein and a conserved coactivator protein (Mastermind family in mammals and *Drosophila*, SEL-8/LAG-3 in nematodes), and displaces a corepressor complex. The NICD/CSL activator complex up-regulates transcription of target genes whose identity depends on cell type.

**Figure 1:**
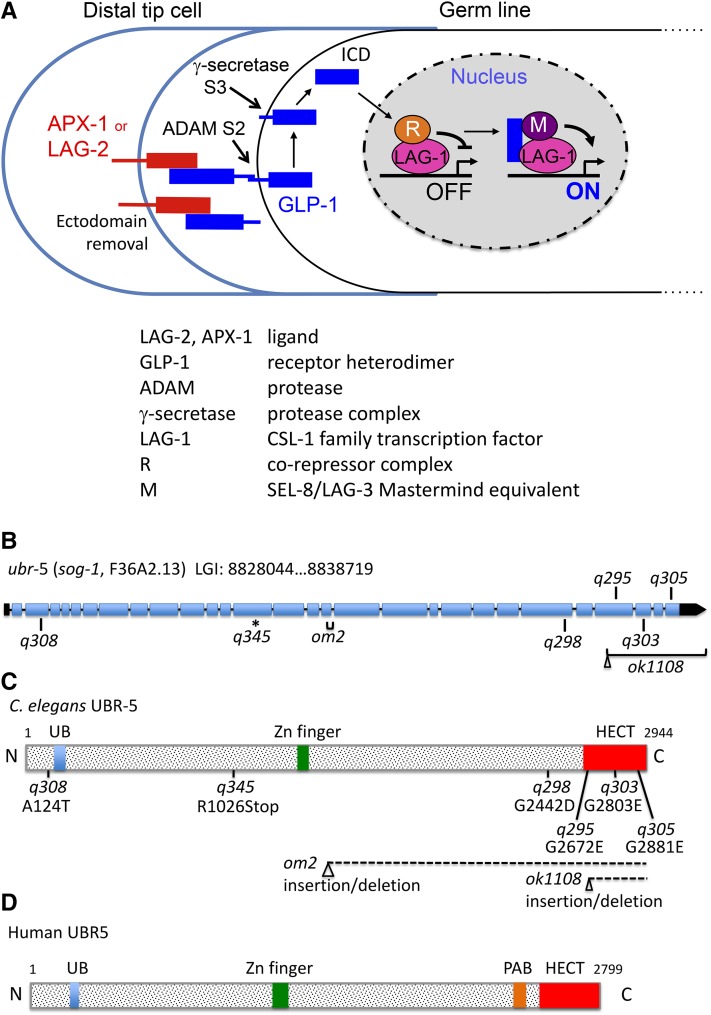
*sog-1* encodes UBR-5, a HECT-type E3 ubiquitin ligase. (A) Working model for GLP-1/Notch signaling in the adult *C. elegans* germline. Interaction of transmembrane LAG-2 and/or APX-1 ligand and GLP-1 heterodimer triggers proteolytic cleavage of GLP-1. The S2 cleavage requires an ADAM family protease and releases the GLP-1 ectodomain (bound to LAG-2); the S3 cleavage requires γ-secretase and releases the GLP-1 intracellular domain (ICD) for transport to the nucleus. Nuclear GLP-1 ICD interacts with the CSL-1-type transcription factor, LAG-1, and the coactivator SEL-8/LAG-3 (M), and displaces the LAG-1-bound corepressor complex (R). Signaling “strength” is modulated by numerous processes, as described in the text. (B) Diagram represents *ubr-5* gene structure; mutant lesions associated with suppression of *glp-1* are indicated. Nucleotide coordinates refer to genome version WS240. (C) Diagram represents UBR-5 protein structure. Conserved domains are indicated, as are amino acid substitutions and deletions/insertions associated with mutant alleles. The UB domain is variably referred to as the EDD or E3 domain in the literature. See Table S2 for details of sequence insertions and deletions in *ok1108* and *om2*. (D) Domain architecture of human UBR5. Many UBR5 family members contain a poly(A) binding protein (PABP) motif (aka MLLE motif) just upstream of the HECT domain.

Notch-type signaling is modulated by a variety of mechanisms (Louvi and Artavanis-Tsakonas 2012; Yamamoto *et al.* 2014). In the signaling cell, endocytosis of the ligand−receptor complex causes an essential conformational change that allows the S2 cleavage to occur. This process requires components of the endosomal trafficking machinery and mono-ubiquination of DSL intracellular domain by E3 ubiquitin ligases. In some signaling contexts, endocytosis also plays an earlier role in activation of ligand. In the receiving cell, newly synthesized Notch-type receptor receives a variety of post-translational modifications that modulate its activity prior to reaching the cell surface. As is the case for signaling activity in general, it is important that Notch-type signaling not be continuous, and mechanisms are in place to down-regulate NICD activity (reviewed by Baron 2012; Barth and Kohler 2014; Le Bras *et al.* 2011; Kandachar and Roegiers 2012; Weinmaster and Fischer 2011). For example, NICD activity is limited by ubiquitin-mediated targeting to the proteasome for degradation (reviewed by Lai 2002; Wang *et al.* 2011). In this process, ubiquitin is covalently linked to ubiquitin-activating enzyme (E1), transferred to ubiquitin-conjugating enzyme (E2), and finally transferred to the protein substrate (*e.g.*, NICD) via the activity of a ubiquitin–protein ligase (E3) (reviewed by Kipreos 2005; Hershko and Ciechanover 1998). Target specificity is conferred by the E3 ligase, and SEL-10/Fbw7 is a conserved E3 ligase component that limits Notch signaling activity in many species (Hubbard *et al.* 1997; see reviews by Lai 2002; Wang *et al.* 2011). In addition to promoting protein turnover, E3-mediated ubiquitination can modulate proteinprotein interactions and impact processes such as nuclear import (Rodriguez 2014). Therefore, E3 ligase activity may also regulate Notch signaling by modulating interactions between signaling components.

Two Notch isoforms are present in *Caenorhabditis elegans*: GLP-1 (germline proliferation defective-1) and LIN-12 (lineage defective-12) (Greenwald and Kovall 2013). GLP-1 and/or LIN-12 mediate numerous cell-signaling events throughout development and in the adult gonad (reviewed by Priess 2005; Sternberg 2005; Greenwald and Kovall 2013). GLP-1 is active in cell−cell interactions in the embryo and in soma-to-germline signaling in the larval and adult gonad. LIN-12 is redundant with GLP-1 for some signaling events in the late embryo and later mediates signaling events in many different somatic cells during larval development. Numerous modulators of Notch signaling – as well as core components of the pathway – have been identified in extensive genetic screens for suppressors or enhancers of loss- or gain-of-function mutations in *glp-1*, *lin-12*, or components of the γ-secretase complex (reviewed by Greenwald and Kovall 2013). Most of these modulators also regulate additional developmental processes not known to involve Notch signaling.

GLP-1-mediated inductive signaling from the somatic gonad to the germline is essential for germ cell proliferation in the larva and adult (reviewed by Hansen and Schedl 2013; Kimble and Seidel 2013). All germ cells are proliferative in early larval development; later, proximal germ cells enter meiosis, and germline proliferation becomes restricted to the distal region of the gonad arm. During the early proliferative phase, several somatic gonadal cells signal to the germline via GLP-1; in the later phase, only the distal tip cell signals to the germline via GLP-1. Proliferative germ cells are not maintained if signaling is reduced or abolished by mutation in a GLP-1 pathway component or by ablation of somatic signaling cells; instead, germ cells prematurely exit mitosis, enter meiosis, and form gametes. Additional factors promote a wild-type level of germ cell proliferation, including the distal sheath cells (Killian and Hubbard 2005), gap junctions between the somatic gonad and germline (Starich *et al.* 2014), and nutritional factors, among others (Hubbard *et al.* 2013).

We previously recovered suppressor of *glp-1* (*sog*) mutations in genetic screens for suppressors of the *glp-1* temperature-sensitive (*ts*) phenotype (Maine and Kimble 1993). The *sog* mutations partially suppressed *glp-1* maternal effect embryonic lethality and germline proliferation defects. Here, we report the molecular characterization of *sog-1*. We demonstrate that *sog-1* encodes UBR-5, a member of the HECT (homologous to the E6-AP carboxyl terminus)-type E3 protein–ubiquitin ligase family whose closest mammalian relative is UBR5 (Ubiquitin protein ligase E3 component n-recognin 5; also called EDD, E3 identified by differential display). We find that loss of UBR-5 activity causes an increase in both GLP-1 and LIN-12 signaling activity in sensitized genetic backgrounds where the receptor carries a conditional mutation. Genetic analysis suggests that UBR-5 acts in the receiving cell. The SCF^SEL-10^ E3 ubiquitin−ligase complex is known to limit LIN-12 activity and, to a minor extent, GLP-1 activity by binding to and promoting turnover of the intracellular domain (Sundaram and Greenwald 1993; Hubbard *et al.* 1997). Our genetic analysis suggests that UBR-5 and SEL-10 function in concert to limit Notch signaling in some tissues.

## Materials and Methods

### Mutant strains

*C. elegans* strains were cultured using standard methods (Epstein and Shakes 1995). All strains were derived from the Bristol strain, N2. Mutations used in this study are described in www.wormbase.org unless otherwise noted and include the following:

LGI: *sog-1* alleles *q295*, *q298*, *q303*, *q305*, *q308*, *q345* (all described in Maine and Kimble 1993), *om2* (this study), *ok1108* (OMRF Knockout Group, see wormbase.org), *rrf-1(pk1417)*, *unc-13(e51)*, *nDf25*, *ccIs4251 [myo-3p*::*GFP(NLS)*::*LacZ (pSAK2) + myo-3p*::*GFP* (mitochondrially targeted) (pSAK4) + *dpy-20(+)]*.
LGIII: *glp-1(q231ts)*, *glp-1(ar202ts)*, *lin-12(ar170)*, *unc-32(e189)*.
LGIV: *lag-1(om13ts)*, *nT1 [qIs51]*.
LGV: *him-5(e1467)*, *him-5(e1490)*, *sel-10(ok1632)*.

In addition, *syIs50 [cdh-3*::*GFP* + *dpy-2(+)]* served as an anchor cell marker (Pettitt *et al.* 1996; Inoue *et al.* 2002).

The *om2* deletion allele was isolated in a noncomplementation screen as follows. L4 *unc-13(e51)*; *glp-1(q231)* hermaphrodites were treated with 20−30 μg/ml trimethylpsoralen (TMP) in M9 medium, irradiated for 20 sec at a distance of 10 cm with a long-wave UV power source, allowed to recover for several hr, and mated with *sog-1(q298);glp-1(q231);him-5(e1490)* males at 15°. Mating plates were shifted to 20°, after a substantial number of F1 embryos had been produced, and screened 2−3 d later for the presence of fertile non-Unc cross-progeny, which were presumed to be genotype *unc-13(+) sog-1(q298)/unc-13 sog-1(omx)*; *glp-1(q231)*; *him-5(e1490/+)*. Homozygous *unc-13 sog-1(omx)*; *glp-1(q231)* animals were recovered and out-crossed to confirm that the new *sog* allele was linked to *unc-13*.

### Whole genome sequencing

Genomic DNA isolation from strains JK946 [carrying *sog-1(q303)*] and JK952 [carrying *sog-1(q308)*], library construction, whole genome sequencing (WGS), and bioinformatics analysis were performed as described (Flibotte *et al.* 2010; Thompson *et al.* 2013). For analysis of other *sog-1* strains, the F36A2.13 gene region was recovered by DNA amplification and sequenced using standard methods. The *sog-1* mutations were originally mapped to the cluster on LGI, and we particularly focused on WGS data from this region.

In the course of this study, we also performed WGS analysis of JK953, a strain previously reported to carry a *sog-1* mutation called *q309*. Unlike other *sog-1* alleles, which were recovered in F2 screens for recessive suppressors of *glp-1*, *q309* was recovered in the course of a dominant suppressor screen as described (Maine and Kimble 1993). WGS analysis revealed that JK953 is wild-type for *glp-1* and does not contain a mutation in F36A2.13. We conclude that the reduced brood size and temperature sensitivity associated with JK953 result from a combination of other mutations in the genetic background.

**Figure 2:**
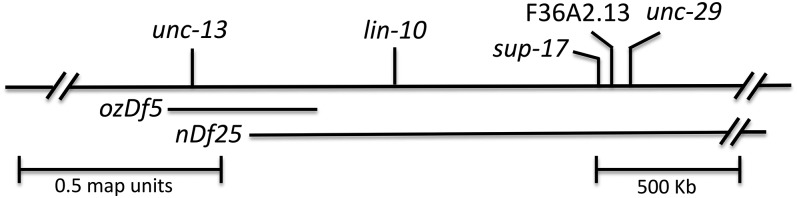
Genetic and physical map of the *ubr-5* region. Genetic mapping previously placed *sog-1* between *unc-13* and *lin-10*. Our molecular studies reported here indicate that *sog-1* corresponds to F36A2.13/*ubr-5*, located between *lin-10* and *unc-29*. The positions shown for deficiencies *ozDf5* and *nDf25* reflect their ability to uncover mutations in genes in the region.

Our molecular data led us to revise how we interpret earlier gene dosage data. Genetic mapping had placed *sog-1* mutations in a region uncovered by the deficiencies *ozDf5* and *nDf25* (Maine and Kimble 1993). Neither *sog-1(-)/ozDf5* nor *sog-1(-)/nDf25* suppressed *glp-1(q231)*; therefore we speculated that the *sog-1* alleles isolated in our *glp-1* suppression screen were recessive gain-of-function mutations. Now, based on our molecular identification of *sog-1* as located outside the region uncovered by *ozDf5*, we would not expect *sog-1/ozDf5* to suppress *glp-1(ts)* (Figure 2). In contrast, *nDf25* uncovers genes that flank *sog-1*, suggesting it should uncover *sog-1* (Figure 2). To investigate further, we generated an *nDf25/unc-13 ccIs4251*; *glp-1(q231ts)* strain and mated hermaphrodites of this genotype with *sog-1(om2);glp-1(q231ts)* males to generate *nDf25/sog-1(om2)*; *glp-1(q231ts)* animals. This assay is straightforward compared with our earlier test because use of a GFP-tagged chromosome obviates the need for other marker mutations and allows us to identify unambiguously the very slow growing *nDf25/sog-1(om2)* cross-progeny. Matings were conducted at 15°, and progeny were shifted to 20° after hatching. *nDf25/sog-1(om2)*; *glp-1(q231ts)* hermaphrodites were picked to a separate plate and observed to segregate viable embryos, indicating that *nDf25/sog-1(om2)* suppresses *glp-1(q231ts)*. Hence, *nDf25* indeed appears to uncover *sog-1*. *nDf25/sog-1(om2)*; *glp-1(q231ts)* hermaphrodites also segregated nonviable embryos, presumably *nDf25* homozygotes.

### Phenotypic analysis

Brood size assays were carried out using standard methods as follows. All *sog-1;unc-32 glp-1(231)* strains were maintained at 20°. The *unc-32 glp-1(q231ts)* control strain was maintained at 15°, and animals to be used for brood size experiments were shifted to 20° as late-stage embryos or newly hatched L1 larvae. Broods were assayed by placing individual L4 larvae onto single plates; once they became gravid adults, they were moved to a fresh plate daily until they no longer produced embryos. Embryos were counted immediately after the mother was moved; viable L4 larvae were counted 2−3 d later.

The anchor cell (AC)-ventral uterine (VU) precursor phenotype was assayed by examining late L2 stage larvae with differential interference contrast and epifluorescence microscopy using a Zeiss Axioscope. Anchor cells were identified based on morphology, position within the gonad primordium, and expression of a *cdh-3*::*gfp* transgene included in the strain.

Proliferative zone size was analyzed in adults labeled with DAPI using standard methods (*e.g.*, Qiao *et al.* 1995). L4 stage hermaphrodites were picked to a fresh culture plate, aged 24−25 hr, fixed for ∼15 min with −20° methanol, stained for ∼15 min with 0.2 μg/ml DAPI, and mounted in Vectashield (Vector Laboratories) for epifluorescence analysis. To assay the proliferative region of each specimen, we counted the number of rows of germ cells distal to the leptotene−zygotene “transition” zone.

### RNAi assays

RNA interference (RNAi) was performed by the feeding method (Timmons *et al.* 2001). L4 larvae were placed onto culture plates seeded with *E. coli* expressing *sog-1* double-stranded RNA (dsRNA), and their F1 progeny were assayed in the first day of adulthood for the presence/absence of embryos. Animals were removed from culture plates as they were counted. Non-RNAi controls were performed by culturing each strain [*glp-1(ar202)* or *rrf-1(pk1417);glp-1(ar202)*] on the standard OP50 *E. coli* food source. Presence of the *rrf-1(pk1417)* deletion was verified by DNA amplification. In addition, the sensitivity of *rrf-1(pk1417);glp-1(ar202)* animals to RNAi of various somatic genes was tested to confirm the presence of the RNAi defect as described by Kumsta and Hansen (2012). As a control for an effect of RNAi *per se* on the *glp-1(ar202)* phenotype, RNAi was performed with empty L4440 vector, which produces a short dsRNA that does not correspond to *C. elegans* genomic sequence.

### Data availability

Strains are available upon request. Whole genome sequence data are provided in Supplemental Material, Table S1.

## Results

### sog-1 encodes a HECT-type E3 ubiquitin ligase

Mutations in *sog-1* were recovered in a genetic screen for recessive suppressors of *glp-1(q224ts)* and *glp-1(q231ts)* (Maine and Kimble 1993). Both of these *glp-1(ts)* alleles have a Glp-1 null phenotype at 25° and a partial loss-of-function (*lf*) phenotype at 20° (Maine and Kimble 1989). Consequently, when L1 stage *glp-1(ts)* larvae are shifted from permissive temperature to 25°, their germ cells exit mitosis, enter meiosis, and undergo spermatogenesis. In contrast, when L1 stage *glp-1(ts)* larvae are shifted from permissive temperature to 20°, their germ cells proliferate for a period of time before prematurely entering meiosis and undergoing gametogenesis; in most cases, a full complement of sperm and some oocytes form and some embryos are generated. These embryos die due to defects in GLP-1 signaling during embryogenesis. The *sog-1* mutations partially suppress the Glp-1(ts) defects at 20°, but not at 25°, and therefore do not bypass the requirement for GLP-1 activity (Maine and Kimble 1993).

We initiated a molecular study of *sog-1* by performing whole genome sequence analysis of two *sog-1* mutant strains: JK946, containing *sog-1(q303)*; and JK952, containing *sog-1(q308)* (see *Materials and Methods*). When we compared the JK946 and JK952 sequence data to the reference *C. elegans* genome, we identified numerous common mutations that presumably were present in the original *unc-32(e189) glp-1(q231ts)* strain prior to mutagenesis (Table S1). In addition, we identified a number of mutations unique to either JK946 or JK952. Of note, JK946 and JK952 contain distinct mutations in a common open reading frame (ORF), F36A2.13 (Table S1, Figure 1B). F36A2.13 is predicted to encode a member of the HECT family of E3 ubiquitin ligases (Figure 1). Its closest mammalian relative is UBR5/EDD (Callaghan *et al.* 1998; Tasaki *et al.* 2005), and consequently F36A2.13 is listed in Wormbase as *ubr-5* (UBR E3 ubiquitin ligase homolog – 5) (www.wormbase.org).

We confirmed that *ubr-5* and *sog-1* are the same gene by amplifying and sequencing the F36A2.13 genomic region from five additional strains carrying *sog-1* alleles recovered following mutagenesis with EMS (*q295*, *q298*, *q305*, *q345*; Maine and Kimble 1993) or UV (*om2*; see *Materials and Methods*). Each strain contained a mutation in F36A2.13 (Figure 1, Table S2). In addition, we obtained an F36A2.13 deletion allele, *ok1108*, from the *Caenorhabditis* Genetics Center and tested its ability to suppress *glp-1(q231ts)*. At 20°, *ok1108* partially suppresses *glp-1(q231ts)* (Table 1). We conclude that *sog-1* and F36A2.13 are the same gene. Although *sog-1* is the original published gene name, “*ubr-5*” better denotes the gene product. Therefore, we will refer to F36A2.13 as *ubr-5* for the rest of this article.

**Table 1:** Suppression of *glp-1(q231ts)* by *ubr-5* deletion alleles at 20°

Genotype	Avg. No. Embryos Produced per Brood	% Viable	*n*
*unc-32 glp-1(q231ts)*	129 ± 8*^a^*	0	16
*ubr-5(om2);unc-32 glp-1(q231ts)*	174 ± 10	40	10
*ubr-5(ok1108);unc-32 glp-1(q231ts)*	189 ± 6	34	14

Full broods were counted for the indicated (“*n*”) number of hermaphrodites, including both viable and nonviable embryos.

a The baseline No. of embryos produced by *unc-32 glp-1(q231ts)* controls in these experiments was substantially higher than previously reported (*e.g.*, Maine and Kimble 1989, 1993). Controls were performed with two strains, both of which had been frozen since the early 1990s and were thawed specifically for these assays (see *Materials and Methods*). As described in Maine and Kimble (1993), ∼98% of *unc-32 glp-1(q231ts)* controls produced some (nonviable) embryos, and only ∼2% were Glp-1 sterile. >99.9% of *ubr-5(-);glp-1(q231ts)* animals produced embryos.

Previous analysis of *ubr-5* mutants indicated that they were superficially normal (Maine and Kimble 1993). We reevaluated this question with the deletion alleles, *ubr-5(om2)* and *ubr-5(ok1108)*. These mutants likewise do not have obvious developmental defects in a *glp-1(+)* background under laboratory conditions. In particular, we considered that *ubr-5* mutants might impact the germline stem cell pool. As a measure of proliferative zone size, we counted the number of rows of germ cell nuclei from the distal end of the somatic gonad to the start of the leptotene/zygotene region in animals raised at 20°. When we compared the number of rows of nuclei in the mitotic zone in *glp-1(q231ts)* and *ubr-5(om2);glp-1(q231ts)* animals at 24 hr post-L4 stage, we observed an increase in mitotic zone size from an average of four rows in *glp-1(q231ts)* to an average of 11 rows in *ubr-5(om2)*; *glp-1(q231ts)* (Table 2). Therefore, the loss of UBR-5 activity leads to increased germ cell proliferation in the sensitized GLP-1(ts) background. In contrast, we did not observe an increase in the number of rows of mitotic germ cells in *ubr-5* mutants compared with wild-type at 24 hr post-L4 (Table 2). Hence, the loss of UBR-5 activity did not impact the length of the mitotic region in germlines with wild-type GLP-1 function.

**Table 2:** Suppression of the *glp-1(q231ts)* germline proliferation defect by *ubr-5(om2)*

Genotype	No. Rows of Nuclei in Proliferative Zone*^a^* (Range)	*n*
Wild type (N2)	21 ± 0.6 (16–24)	18
*ubr-5(om2)*	21 ± 0.8 (16–25)	13
*glp-1(q231ts)**^b^*	4 ± 0.7 (0–11)	36
*ubr-5(om2);glp-1(q231ts)*	11 ± 0.4 (5–16)	32
*sel-10(ok1632)**^c^*	15 ± 0.6 (11–20)	14
*glp-1(q231ts)*; *sel-10(ok1632)*	6 ± 0.9 (0–14)	24
*ubr-5(om2);glp-1(q231ts);sel-10(ok1632)*	12 ± 0.6 (8–19)	21
*ubr-5(om2);sel-10(ok1632)*	19 ± 1.2 (13–28)	16

Assays were conducted at 20°. L4 stage larvae were picked to a fresh plate and DAPI-stained 24 hr later. *n*, number of gonad arms evaluated.

a ± represents standard error of the mean. The number of rows of proliferative nuclei was rounded to the nearest whole number.

b The *unc-32(e189) glp-1(q231ts)* strain was maintained at 15°; late-stage embryos were shifted to 20° for growth at restrictive temperature. All *glp-1(q231ts)* strains listed here carry the *unc-32(q231)* marker mutation.

c We note that the *sel-10(ok1632)* strain, RB1432, contains additional mutations that may reduce mitotic zone size. See text.

### UBR-5 negatively regulates GLP-1 activity

The predicted *ubr-5* product is a 2944 amino acid protein with domain architecture characteristic of the UBR5/EDD HECT protein subfamily. It contains three conserved motifs: a UB (also called E3) domain located near the N terminus, a zinc-finger domain located in the middle of the protein (the UBR motif), and a C-terminal HECTc domain (Figure 1C). Many members of this protein family also contain a PABP (poly A binding protein; also called MLLE) domain within ∼100 amino acids of the HECT domain (Callaghan *et al.* 1998; Tasaki *et al.* 2005; Scheffner and Kumar 2014) (Figure 1D). *C. elegans* UBR-5 appears to lack this domain. Among the eight *ubr-5* alleles we characterized, three contain a premature stop codon. The *ubr-5(om2)* allele is likely to be null; it contains a deletion of 105 nucleotides in the center of the gene just downstream of the zinc-finger domain that is predicted to shift the ORF, inserting 45 amino acids and deleting the C-terminal half of the protein, including the entire HECT domain (Figure 1, Table S2). Similarly, *ubr-5(q345)* may be null as it contains a single nucleotide change that converts residue 1026 to a stop codon (Figure 1, Table S2). It is predicted to encode a truncated protein lacking the zinc-finger and HECT domains. *ubr-5(ok1108)* is a complex mutation with a 1360 nucleotide deletion and 73 nucleotide insertion; most of the HECT domain is deleted as well as the 3′ UTR and some downstream sequence (Figure 1, Table S2). The net result of the mutation is to insert nine amino acids downstream of residue 2631. This allele is expected to lack E3 ligase activity, as well. The nature of these alleles suggests that a reduction in *ubr-5* function suppresses the loss of *glp-1* activity, and therefore UBR-5 is a negative regulator of GLP-1. The other *ubr-5* alleles we characterized contain missense mutations predicted to cause single amino acid substitutions as follows: *q308* just upstream of the E3 domain; *q298* just upstream of the HECT domain; and *q295*, *q303*, and *q305* within the HECT domain (Figure 1, Table S2).

We characterized *glp-1* suppression by the *ubr-5* deletion alleles, *om2* and *ok1108* (Table 1), and compared the results with data obtained previously for other alleles (Maine and Kimble 1993). We evaluated the total number of progeny (viable and nonviable) produced and the number of progeny that hatched and developed to adulthood. As observed previously (Maine and Kimble 1993), we do not see a simple relationship between suppression of the brood size defect and embryonic lethality, and the likely null alleles do not show identical suppression. We suspect that suppression is influenced by other, unique mutations present in different *ubr-5* strains. For example, WGS data revealed a suite of shared mutations and a number of unique mutations in *ubr-5(q303)*; *unc-32(e189) glp-1(q231)* and *ubr-5(q308)*; *unc-32(e189) glp-1(q231)* (Table S1). We hypothesize that some of these mutations may influence the degree of suppression by *ubr-5* in one or more tissues. Indeed, it was noted during previous three-factor mapping experiments that suppression by *ubr-5* is abrogated to a large extent in the presence of certain marker mutations (Maine and Kimble 1993). As detailed in the *Materials and Methods*, data obtained in the course of our studies allow us to make corrections to the literature with respect to (i) gene dosage requirements and (ii) the identity of a previously reported allele, *q309*.

To confirm further that the *ubr-5* mutations are loss-of-function, we knocked down UBR-5 protein in a *glp-1* gain-of-function background using RNAi and examined the consequences for GLP-1 signaling. Gain-of-function *glp-1* mutations have elevated GLP-1 signaling, resulting in germline over-proliferation and eventual formation of a germline tumor (Berry *et al.* 1997; Pepper *et al.* 2003). The *glp-1(ar202gf)* allele is temperature sensitive, producing a more elevated level of GLP-1 signaling at higher temperatures (Pepper *et al.* 2003). At 25°, germ cells form a tumor relatively early in development, and the animal does not produce oocytes. At lower (semipermissive) temperatures, overproliferation occurs more slowly, and a sizable proportion of *glp-1(ar202)* mutants are fertile (Pepper *et al.* 2003).

**Figure 3:**
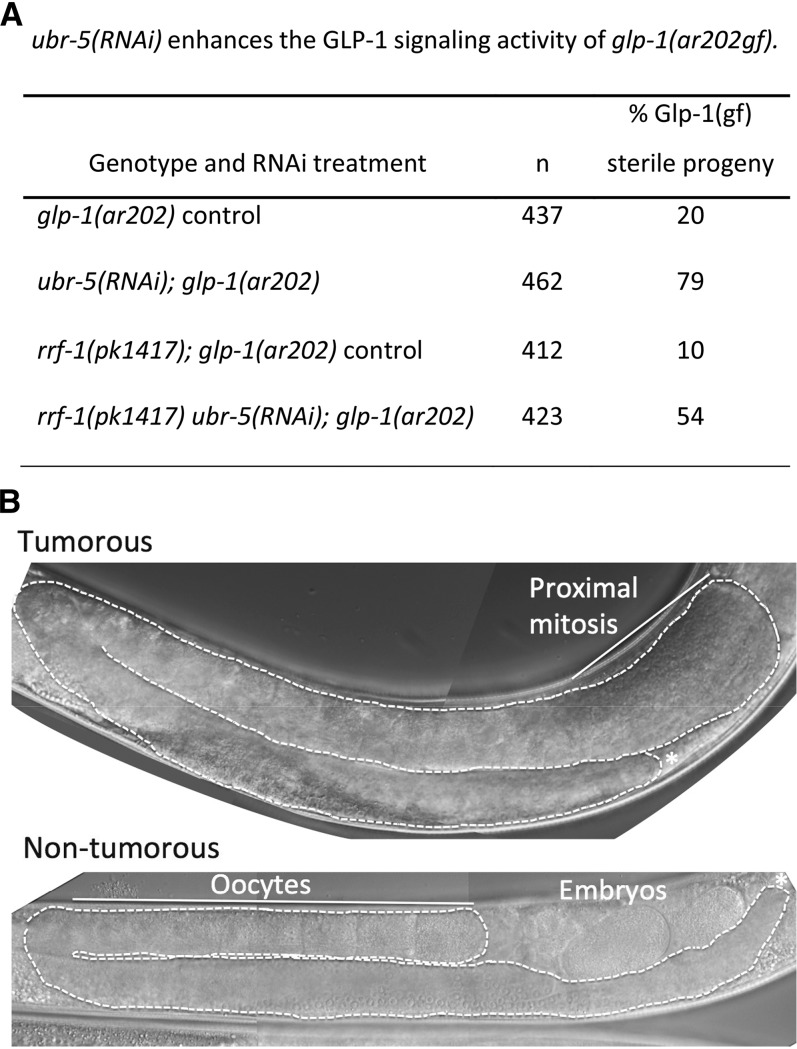
*ubr-5(RNAi)* enhances *glp-1(ar202gf)*. (A) Assays were performed at 22°, a semipermissive temperature for *glp-1(ar202gf)*. L4 larvae were placed onto *ubr-5(RNAi)* or control plates, and their adult progeny were evaluated for fertility or sterility. Three replicate sets of experiments were performed, where all four treatments were run in parallel. In the *rrf-1(+)* background, *ubr-5* RNAi is active in all tissues; in the *rrf-1(0)* background, *ubr-5* RNAi is active in the germline, but not in the somatic gonad (Kumsta and Hansen 2012). The Glp-1 *gf* phenotype is significantly enhanced in both *rrf-1(+)* and *rrf-1(0)* backgrounds relative to the appropriate non-RNAi control assay (animals cultured on OP50 bacteria). A paired *t*-test indicates no difference between enhancement of *glp-1(ar202gf)* in *rrf-1(+)* and *rrf-1(0)* samples. In contrast to the 4- to 5.5-fold increase in % tumorous animals upon *ubr-5(RNAi)*, negative controls performed with “empty vector” (L4440 plasmid without an insert) increased the % tumorous animals by 0.4- to 0.5-fold relative to controls grown in parallel [*n* = 449 *glp-1(ar202)*, *n* = 78 *rrf-1;glp-1(ar202)*]. *n*, total number of animals scored in replicate treatments. (B) Examples of the *glp-1(ar202gf)* Tumorous and non-Tumorous phenotypes observed under our assay conditions.

We tested the extent to which reducing *ubr-5* activity would increase the level of GLP-1 signaling in the *glp-1(ar202)* background at the semipermissive temperature, 22°. We assayed the increase in GLP-1 signaling by quantifying the increase in % tumorous sterility in the population at ∼24 hr post-L4 stage. Under conditions used in our assay, on average ∼20% of *glp-1(ar202)* control animals were tumorous and lacked embryos, and this phenotype increased nearly fourfold to ∼79% in *ubr-5(RNAi)*; *glp-1(ar202)* animals (Figure 3). The tumorous *glp-1(ar202)* controls and *ubr-5(RNAi)*; *glp-1(ar202)* double mutants contained mitotic nuclei in the proximal germline (Figure 3B). We conclude that *ubr-5(RNAi)* significantly increases the level of GLP-1 signaling in *glp-1(ar202)* mutants. This result supports the conclusion that reduced UBR-5 activity leads to elevated GLP-1 signaling activity.

### UBR-5 acts in the germ cells to limit GLP-1 pathway activity in the gonad

To evaluate whether UBR-5 activity is important in the signaling and/or receiving cell, we assayed the ability of *ubr-5(RNAi)* to enhance GLP-1 signaling activity in an *rrf-1(0)* mutant background. In *rrf-1(pk1417)* mutants, RNAi is severely impaired in many somatic tissues, including the somatic gonad (Kumsta and Hansen 2012; Sijen *et al.* 2001). We performed *ubr-5* RNAi in parallel in *rrf-1(pk1417);glp-1(ar202)* and *rrf-1(+)*; *glp-1(ar202)* hermaphrodites and assayed enhancement of the *glp-1(ar202gf)* sterile phenotype (Figure 3). *glp-1(ar202gf)* sterility was enhanced significantly in *rrf-1(pk1417)* animals (Figure 3). Statistical analysis indicates no significant difference in the degree of enhancement in the *rrf-1(+)* *vs.* *rrf-1(pk1417)* backgrounds; therefore we conclude that UBR-5 acts in the germline to limit GLP-1 signaling and germ cell proliferation.

### UBR-5 negatively regulates LIN-12 activity

As a result of LIN-12-mediated lateral signaling between cells in the L2 stage gonad primordium, Z1.ppp and Z4.aaa, one cell becomes the AC and the other cell becomes a VU precursor cell. In the absence of LIN-12 activity, both Z1.ppp and Z4.aaa take on an AC fate (Greenwald *et al.* 1983; Seydoux and Greenwald 1989). To determine if UBR-5 activity limits signaling via LIN-12/Notch, we evaluated the ability of *ubr-5(om2)* and *ubr-5(q345)* to suppress the 2-AC phenotype in *lin-12(ar170ts)* mutants at 25°. We included a *cdh-3*::*gfp* transgene in our strains to aid in identification of ACs (Karp and Greenwald 2003). CDH-3 is a cadherin protein expressed by the AC but not by other nearby cells during late L2/early L3 stage (Pettitt *et al.* 1996). At 25°, we observed two ACs in ∼64–68% of *lin-12(ar170)* controls scored at late L2 stage (Table 3). In contrast, we observed two ACs in ∼33% of *ubr-5(om2)*; *lin-12(ar170)* and *ubr-5(q345)*; *lin-12(ar170)* larvae grown in parallel with controls (Table 3). This statistically significant reduction in the 2-AC phenotype indicates that *ubr-5(om2)* and *ubr-5(q345)* partially suppress the loss of *lin-12* function. We conclude that wild-type UBR-5 activity limits signaling via the LIN-12 receptor in the Z1.ppp–Z4.aaa interaction.

**Table 3:** Loss of *ubr-5* function suppresses the *lin-12* 2-AC defect

Strain	% 2 AC	*n*
*unc-32(e189) lin-12(ar170)*; *cdh-3*::*gfp*	67.6	108
*ubr-5(om2)*; *unc-32(e189) lin-12(ar170)*; *cdh-3*::*gfp*	33.3	96
*unc-32(e189) lin-12(ar170)*; *cdh-3*::*gfp*	63.6	110
*ubr-5(q345)*; *unc-32(e189) lin-12(ar170)*; *cdh-3*::*gfp*	33.0	100

Assays were conducted at 25°. Control *unc-32(e189) lin-12(ar170)*; *cdh-3*::*gfp* animals were assayed in parallel with each *ubr-5(-);unc-32(e189) lin-12(ar170);cdh-3*::*gfp* strain. In each case, the value for “% 2 AC” was significantly different in control *vs.* experimental strains, *P* < 0.03 (Z-test). *n*, number of larvae evaluated; AC, anchor cell.

### ubr-5 mutations do not suppress lag-1(ts) lethality

We hypothesize that UBR-5, as an E3 ligase, may limit GLP-1 signaling by promoting turnover of a pathway component, *e.g.*, full-length GLP-1 or processed GLP-1 intracellular domain, or a positive modulator of pathway activity. By this scenario, an elevated level of the partially active GLP-1(q231) protein would accumulate in *ubr-5* mutants and GLP-1 signaling activity would increase. To investigate potential UBR-5 targets, we tested whether *ubr-5* mutations can suppress a *ts* mutation in the Notch pathway transcription factor, LAG-1. The *lag-1(om13ts)* mutation strongly enhances the *glp-1(bn18ts)* germline proliferation defect at semipermissive temperatures and, in a *glp-1(+)* background, causes embryonic and early larval lethality (Qiao *et al.* 1995). Larval lethality occurs at the L1 stage shortly after hatching and is characteristic of the Lag phenotype (Lambie and Kimble 1991). CSL-type transcription factors also function independently of Notch (reviewed by Johnson and MacDonald 2011; Ghai and Gaudet 2008), and we hypothesize that at least some aspects of the *lag-1(om13ts)* phenotype may be independent of GLP-1 and LIN-12 signaling.

We evaluated the ability of *ubr-5(ok1108)* to suppress the *lag-1(om13ts)* embryonic and larval defects at 20°. The *lag-1(om13ts)* single mutant and *ubr-5(ok1108);lag-1(om13ts)* double mutant strains had similar viability at 20° (4–5%; Table 4). In addition, brood sizes were similar for the two strains, and *ubr-5(ok1108)* did not appreciably change the proportion of progeny that died as embryos or as L1 stage larvae (Table 4). We interpret these data to indicate that *ubr-5* does not suppress the *lag-1(om13ts)* embryonic or larval lethality.

**Table 4:** Loss of *ubr-5* function does not suppress *lag-1(ts)* phenotypic defects

Genotype	Avg No. Embryos Produced (± SE)	% Dead Embryos	% Dead Larvae	% Viable Progeny	*n*
*lag-1(om13ts)*	188 ± 18	39.6	57.2	3.2	6
*ubr-5(ok1108);lag-1(om13ts)*	225 ± 11	48.6	47.7	3.7	10

Tests were performed at 20°. Most nonviable larvae died at early L1 stage, as is characteristic of the Lag phenotype (Lambie and Kimble 1991). *n*, number of full broods counted.

### ubr-5 and sel-10 interact synergistically to suppress glp-1 embryonic lethality

SEL-10 is the F-box component of an SCF (Skp1-Cullin-F-box) E3 ubiquitin–protein ligase complex containing SKR-1 as the Skp1 ortholog. SEL-10 and SKR-1 have been shown independently to limit Notch signaling (Sundaram and Greenwald 1993; Hubbard *et al.* 1997; Killian *et al.* 2008). Members of the SEL-10 family also negatively regulate Notch signaling in other organisms (reviewed by Lai 2002). SEL-10 directly binds the LIN-12 and GLP-1 intracellular domains and is hypothesized to promote their turnover (Hubbard *et al.* 1997). Sundaram and Greenwald (1993) reported that *sel-10(ar41)* very weakly suppressed the *glp-1(q231ts)* maternal effect lethality at 20°, producing an average of 1.5 viable progeny per hermaphrodite. At 25°, *sel-10(ar41)* did not suppress the *glp-1(q231ts)* germline proliferation defect to an appreciable extent. It should be noted that their assay was performed with a *glp-1(q231ts);sel(arX) sel-10(ar41)* strain, which contained a linked, uncharacterized suppressor, *sel(arX)*, that may have contributed to the suppression phenotype.

We asked whether SEL-10 might be partially redundant with UBR-5 with respect to limiting Notch-type signaling activity. To do so, we evaluated suppression of *glp-1(q231ts)* by *ubr-5* and *sel-10* alone and in combination. *sel-10(ar41)* contains a premature stop codon at residue 323 and is predicted to encode a truncated protein (Hubbard *et al.* 1997). In our experiments, we used *sel-10(ok1632)*, which contains a deletion/insertion close to the 5′ end of the ORF that is predicted to remove all but the first 18 amino acids of SEL-10 (Killian *et al.* 2008). As previously reported for *sel-10(ar41)*, we find that *sel-10(ok1632)* very weakly suppresses the *glp-1(q231ts)* embryonic lethality at 20°, resulting in <1% viability (Table 5). Interestingly, when we examine the *ubr-5(om2);glp-1(q231ts);sel-10(ok1632)* triple mutant, we observe substantially higher offspring viability compared to *ubr-5(om2);glp-1(q231ts)* despite the poor suppression by *sel-10* alone (Table 5). Embryonic viability was ∼75% for *ubr-5(om2);glp-1(q231ts);sel-10(ok1632)* triple mutants as opposed to ∼40% for *ubr-5(om2);glp-1(q231ts)* and <1% for *glp-1(q231ts);sel-10(ok1632)* double mutants.

**Table 5:** Tests for redundancy between UBR-5 and SEL-10

Genotype	Avg No. Embryos Produced (± SE)	*N*	% Viable Progeny	*n*
*ubr-5(om2)*; *unc-32(e189) glp-1(q231ts)*; *sel-10(ok1632)*	124 ± 5	10	74.8	1243
*ubr-5(om2)*; *unc-32(e189) glp-1(q231ts)*	174 ± 10	10	40.0	1740
*unc-32(e189) glp-1(q231ts)*; *sel-10(ok1632)**^a^*	119 ± 15	12	0.6	1428
*unc-32(e189) glp-1(q231ts)**^b^*	129 ± 8	16	0.0	2065
*ubr-5(om2)*; *sel-10(ok1632)**^a^*	248 ± 6	12	97.2	2972
*ubr-5(om2)*	276 ± 17	7	98.4	1901
*sel-10(ok1632)* original RB1432*^a^**^,^**^c^*	157 ± 17	8	32.0	1441
*sel-10(ok1632)* reisolated from *ubr-5(om2)*; *sel-10(ok1632)**^c^*	225 ± 7	5	98.6	1125

Assays were conducted at 20°. *N*, number of full broods counted; *n*, number of individuals counted.

a The reported broods were produced by animals with a functional vulva. Some animals of these genotypes have a defective vulva and consequently fail to lay eggs and/or die prematurely, in each case producing a limited number of offspring that does not reflect the degree of germline proliferation. Hence, the effective brood size of this strain is smaller than the value listed here.

b These data also are listed in Table 1.

c The embryonic lethality and reduced brood size of strain RB1432 do not appear to be caused by *sel-10(ok1632)*. See text.

We also observe a very weak suppression of the germline proliferation defect by *sel-10(ok1632)*. When we compare the average mitotic zone size at 24 hr post-L4 stage, we observe an average of six rows of proliferative nuclei in *glp-1(q231ts);sel-10(ok1632)* compared with four rows in *glp-1(q231ts)* alone (Table 2). Unlike the case for embryonic viability, analysis of mitotic zone size and brood size suggested that *sel-10* and *ubr-5* do not interact synergistically to suppress the *glp-1(q231ts)* germline proliferation defect. Mitotic zone size in the *ubr-5(om2);glp-1(q231ts);sel-10(ok1632)* triple mutant and the *ubr-5(om2);glp-1(q231ts)* double mutant are essentially the same (12 ± 0.6 rows *vs.* 11 ± 0.4 rows of mitotic germ cells) (Table 2). Moreover, *ubr-5(om2);glp-1(q231ts);sel-10(ok1632)* triple mutants produce approximately the same number of embryos as do *glp-1(q231ts);sel-10(ok1632)* double mutants and *glp-1(q231ts)* controls, and substantially fewer embryos than *ubr-5(om2);glp-1(q231ts)* double mutants (Table 5).

In the course of our work, we observed that the *sel-10(ok1632)* strain we obtained from the *Caenorhabditis* Genetics Center, RB1432, has highly penetrant defects not reported for *sel-10* in the literature, including substantial embryonic lethality and a reduced brood size of ∼160 (Table 5). When we examined the *ubr-5(om2);sel-10(ok1632)* double mutant phenotype, we noted that embryonic viability and brood size approximated that of *ubr-5(om2)* single mutants (Table 5). To determine whether *ubr-5(om2)* suppressed these defects or, alternatively, they might be caused by mutations in the RB1432 strain background and were eliminated when we constructed the *ubr-5(om2);sel-10(ok1632)* double, we reisolated the *sel-10(ok1632)* allele from the *ubr-5(om2);sel-10(ok1632)* strain and examined the phenotype. We found that the brood size and % viability of the reisolated *sel-10(ok1632)* single mutant were essentially the same as the *ubr-5(om2);sel-10(ok1632)* double (Table 5). We conclude that strain RB1432 indeed carries one or more additional mutation(s) distinct from *sel-10(ok1632)* causing embryonic lethality and reduced brood size.

## Discussion

Here, we demonstrate that the *sog-1* gene, previously shown to interact genetically with *glp-1*, encodes UBR-5, the sole *C. elegans* member of a conserved E3 ubiquitin ligase family. Mammalian UBR5 and the *Drosophila* ortholog, *hyperplastic discs* (*hyd*), function in diverse aspects of development including cell proliferation (reviewed by Shearer *et al.* 2015; Mansfield *et al.* 1994). Our data indicate that UBR-5 activity limits GLP-1/Notch signaling in the embryo and larval/adult gonad and LIN-12/Notch signaling in the AC/VU decision. Moreover, UBR-5 acts autonomously to repress germline proliferation, suggesting its primary impact on Notch signaling is within the receiving cell. In contrast, UBR-5 activity does not suppress the brood size or viability defects associated with reduced function of LAG-1, the CSL-type transcription factor component of the GLP-1 signaling pathway in the germline. Given these findings, we hypothesize that UBR-5 functions in the turnover of GLP-1/LIN-12 receptor and/or other proteins responsible for receptor production. Alternatively, UBR-5-mediated ubiquitination may modulate signaling by reducing the ability of GLP-1/LIN-12 to interact with other pathway components, *e.g.*, LAG-1 or LAG-3. Additional experiments will be required to determine if UBR-5 restricts Notch-type signaling in all tissues.

Extensive genetic and molecular analysis has revealed that the outcome of Notch-type signaling is extremely sensitive to the level (“strength”) of signaling activity in a wide variety of contexts (Louvi and Artavanis-Tsakonas 2012; Yamamoto *et al.* 2014). Our data are consistent with these previous observations. Eliminating just one negative regulator of GLP-1 and LIN-12 activity, UBR-5, increased signaling activity sufficiently to rescue moderately severe loss-of-function phenotypes and strongly enhance a weak gain-of-function phenotype.

### The relationship between UBR-5 and SEL-10

**Figure 4:**
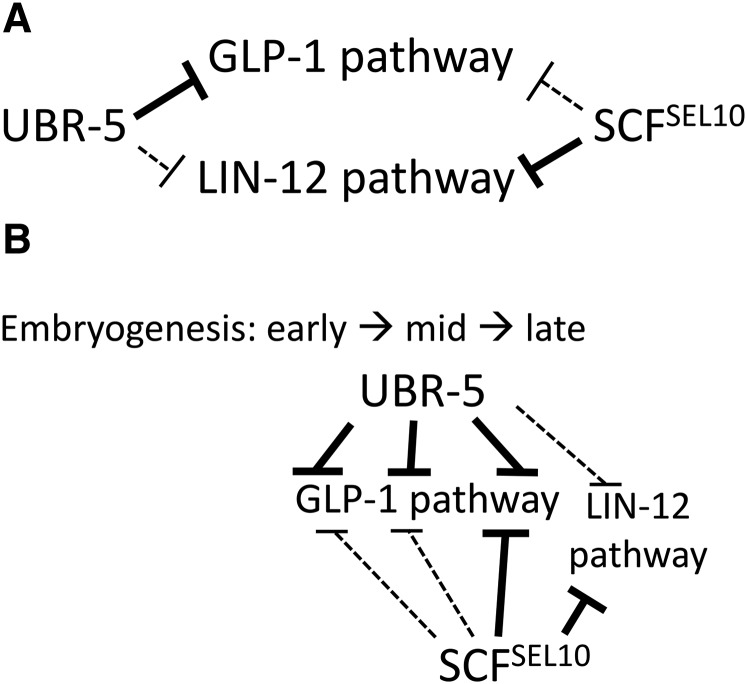
Hypothetical relationships between UBR-5 and SEL-10 activity in the embryo. (A) UBR-5 and SEL-10 may each primarily limit signaling activity via one Notch-type receptor and only play a minor role in limiting signaling via the other receptor. (B) UBR-5 and SEL-10 may limit signaling via both GLP-1 and LIN-12, with UBR-5 having a primary role in the early embryo and both factors acting in the late embryo.

Our data suggest a complex relationship between UBR-5 and SEL-10 with respect to Notch signaling. These findings may reflect, in part, the equivalent receptor activity of GLP-1 and LIN-12 (Fitzgerald *et al.* 1993) and the fact that both GLP-1 and LIN-12 are active in some tissues, *e.g.*, in the embryo, whereas only GLP-1 or LIN-12 is active in other tissues, *e.g.*, GLP-1 in the gonad. Whereas UBR-5 has a major role in limiting Notch signaling in the embryo and gonad, SEL-10 appears to have a larger role in limiting Notch signaling in the embryo than in the gonad. Our observation of a synergistic genetic interaction between *ubr-5* and *sel-10* with respect to suppression of the *glp-1(q231ts)* embryonic lethality indicates that UBR-5 and SEL-10 do not function in a simple linear pathway, at least in embryonic tissues. One model for the relationship between these factors in the embryo is that UBR-5 restricts GLP-1 activity broadly during embryogenesis, whereas SEL-10 restricts GLP-1 primarily in the late embryo – where it also restricts LIN-12 (Figure 4B). Only rarely would development of the *glp-1(q231ts)* embryo be rescued by *sel-10(ok1632)* alone. An alternative hypothesis is that SEL-10 primarily restricts LIN-12 signaling activity and has little impact on GLP-1 signaling activity. This would explain the very minor role of SEL-10 in the gonad, where LIN-12 signaling is not active. One way to think about these data is that UBR-5 may primarily limit GLP-1 signaling, SEL-10 may primarily limit LIN-12 signaling, and reduced UBR-5 or SEL-10 activity may better suppress reduced *glp-1* or *lin-12* activity in those cells or tissues where increased activity of one receptor can compensate for reduced activity of the other, *e.g.*, in the embryo (Figure 4A).

### UBR5 activity in development and disease

UBR5 proteins in mammals and *Drosophila* have been implicated in numerous developmental processes (reviewed by Shearer *et al.* 2015). Vertebrate UBR5 regulates cell cycle progression, and misregulation of UBR5 activity is linked to cancer in many tissues (Scheffner and Kumar 2014; Shearer *et al.* 2015). UBR5 activity appears to promote cell proliferation in some tissues and limit it in others, as the loss of UBR5 activity promotes cancer in some tissues whereas UBR5 overexpression promotes cancer in other tissues. Presumably these differences reflect the multitude of UBR5 targets that may contribute to oncogenesis. The *Drosophila* ortholog, Hyperplastic discs (Hyd), regulates cell proliferation in developing imaginal discs and promotes development of other tissues, as well (Mansfield *et al.* 1994).

UBR5 appears to contribute to cell proliferation control in a number of ways. One role for UBR5 is in modulating activity of the mitotic spindle assembly checkpoint (SAC) mechanism, thereby impacting the ability of cells to enter anaphase. SAC activity ensures cells remain in metaphase until all chromosomes have attached to the mitotic spindle; once chromosomes have done so, then SAC activity must be reduced in order to allow anaphase entry. Evidence suggests that UBR5 functions both to promote SAC activity when needed, *e.g.*, if microtubules are disrupted (Scialpi *et al.* 2015), and to reduce SAC activity once chromosomes have attached to the mitotic spindle (Jiang *et al.* 2015). UBR5 targets different SAC components in these different situations, and its subcellular localization changes during the cell cycle (Scialpi *et al.* 2015; Jiang *et al.* 2015).

Another function of UBR5 is in modulating stability of nuclear myosin 1 (NM1), a factor required for RNA polymerase I transcriptional activity. NM1 is stabilized by GSK3β-mediated phosphorylation, which prevents UBR5-mediated ubiquitination and subsequent degradation (Sarshad *et al.* 2014). Progression beyond G1 stage requires NM1 activity, and hence modulation of NM1 is one means by which UBR5 limits cell cycle progression. Antagonistic activity of GSK3β *vs.* Hyd/UBR5 appears to be a general regulatory mechanism as it also modulates Hedgehog signaling in *Drosophila* (Lee *et al.* 2002; Moncrieff *et al.* 2015). As we observed for Notch signaling, Hedgehog signaling is limited by Hyd/UBR5 activity. Interestingly, mammalian FBW7/SEL10 is recruited to certain targets upon GSK3β−mediated phosphorylation (Flugel *et al.* 2012).

Other studies have implicated UBR5 in modulating the DNA damage response (DDR), where it participates at several steps, and in regulation of certain transcription factors (Shearer *et al.* 2015). One aspect of UBR5 activity is that it limits activity of the DDR machinery to sites of double-strand breaks and prevents inappropriate/unnecessary activity elsewhere on the chromosome. In regulating transcription factor activity, UBR5 functions in many cell types, positively regulating certain transcription factors and negatively regulating others. We present the first evidence, to our knowledge, of a UBR5 family E3 ligase modulating Notch signaling activity. In the future, it will be informative to identify the targets of UBR-5 activity.

## Supplementary Material

Supplemental Material
