# Supplementary material for: UBR-5, a Conserved HECT-Type E3 Ubiquitin Ligase, Negatively Regulates Notch-Type Signaling in Caenorhabditis elegans
Source: G3 (Bethesda). 2016 May 13;6(7):2125–34. doi: 10.1534/g3.116.027805 (PMC4938665; doi:10.1534/g3.116.027805)
Supplement: Supplemental Material [file supp_6_7_2125__index.html]

UBR-5, a Conserved HECT-Type E3 Ubiquitin Ligase, Negatively Regulates Notch-Type Signaling in Caenorhabditis elegans — Supplemental Material 

# UBR-5, a Conserved HECT-Type E3 Ubiquitin Ligase, Negatively Regulates Notch-Type Signaling in *Caenorhabditis elegans*

## Supplemental Material for Safdar *et al.*, 2016

**Files in this Data Supplement:**

- Table S1 - Codon changes detected by whole genome sequence analysis. (.pdf, 434 KB)
- Table S2 - Sequence changes associated with *ubr-5* alleles. (.pdf, 444 KB)
