# Supplementary material for: UBR-5, a Conserved HECT-Type E3 Ubiquitin Ligase, Negatively Regulates Notch-Type Signaling in Caenorhabditis elegans
Source: G3 (Bethesda). 2016 May 13;6(7):2125–34. doi: 10.1534/g3.116.027805 (PMC4938665; doi:10.1534/g3.116.027805)
Supplement: Supplemental Material [file supp_g3.116.027805_TableS1.pdf]

Table S1   Codon changes detected by whole genome sequence analysis

JK946 Chr.	Nucleotide position	Nucleotide change Ref.	JK946	ORF	Gene name	Ref A.A.	JK946 A.A.	Status
I	7092902	C	T	F22D6.5	*prpf-4*	A	T	common
I	7353179	C	G	F07A5.2	F07A5.2	P	A	common
I	8651317	G	A	F39H2.2	*sig-7*	R	K	common
I	8828948	C	T	F36A2.13	F36A2.13	G	E	distinct mutation in JK952
I	13484417	A	G	Y48G10A.2	Y48G10A.2	I	T	common
I	13904800	C	T	Y71A12B.4	*trp-4*	P	S	unique
I	14334390	G	A	F49B2.6	F49B2.6	V	I	common
I	15053241	A	G	F31C3.5	*psf-2*	K	E	common
II	249537	C	A	Y43H11AL.3	*pqn-85*	L	F	unique
II	9864014	C	T	C08H9.4	*chil-1*	A	T	unique
II	9864037	A	G	C08H9.4	*chil-1*	M	T	unique
II	9864061	A	G	C08H9.4	*chil-1*	L	P	unique
II	11468119	T	G	W02B12.8	*rga-1*	L	R	unique
II	11882797	G	C	ZK930.5	ZK930.5	R	T	unique
II	12152415	T	G	Y57A10A.4	Y57A10A.4	D	E	common
II	14165434	G	C	Y48B6A.3	*xrn-2*	D	H	unique
II	14797075	C	A	Y54E2A.11	*eif-3.B*	F	L	unique
II	15026573	G	A	Y53F4B.13	Y53F4B.13	V	I	common
III	15852	G	T	H10E21.1	H10E21.1	D	Y	common
III	2309106	A	G	Y54F10BM.20	Y54F10BM.20	D	G	common
III	5195074	C	A	K10D2.1	K10D2.1	T	K	common
III	5574082	G	A	F09F7.1	*molo-1*	R	Q	unique

III	5864877	T	C	F01F1.4	*rabn-5*	L	S	common
III	6347217	G	A	C56G2.3	C56G2.3	R	Q	unique
III	7325159	G	T	F56C9.11	F56C9.11	P	H	common
III	8440137	C	T	ZK1236.7	*ufbp-1*	G	E	common
III	8888935	C	T	ZK637.14	*unc-32*	M	I	*e189*
III	8975983	C	T	R08D7.6	*pde-2*	G	E	common
III	9098563	G	A	F02A9.6	*glp-1*	G	E	*q231*
III	9201577	G	A	ZC84.1	ZC84.1	P	L	common
III	10111388	C	T	T16H12.9	T16H12.9	P	L	common
III	11630989	A	T	Y66A7A.7	Y66A7A.7	V	D	unique
III	12069789	G	A	Y79H2A.11	*zyg-8*	D	N	unique
III	12219151	C	A	Y75B8A.16	Y75B8A.16	T	K	common
III	12475401	G	A	Y49E10.23	*cccp-1*	A	T	common
III	13398942	C	T	F11F1.1	F11F1.1	A	V	common
IV	595066	G	A	C18H7.4	C18H7.4	D	N	unique
IV	4411894	G	A	Y24D9A.1	*ell-1*	D	N	unique
IV	5042247	C	T	C09B9.8	C09B9.8	G	E	unique
IV	6799139	A	T	C17H12.4	C17H12.4	H	Q	unique
IV	7994439	T	G	Y42H9B.2	*rig-4*	E	A	common
IV	8191095	C	A	C07G1.4	*wsp-1*	D	Y	unique
IV	11445850	G	A	C10C6.1	*kin-4*	V	I	unique
IV	11918668	G	A	ZK822.1	ZK822.1	L	F	unique
V	2200280	C	T	Y5H2B.5	*cyp-32B1*	G	D	unique
V	4982300	T	C	C13D9.6	*srsx-13*	D	G	common
V	7142145	A	C	F10D2.6	*ugt-37*	L	R	common
V	7220103	A	T	C13F10.6	C13F10.6	L	H	common

V	7892362	C	A	F40A3.6	F40A3.6	R	I	common
V	8760428	T	C	B0507.10	B0507.10	K	E	common
V	14786180	C	T	C48G7.3	*rin-1, tag-333*	M	I	common
V	14997197	C	A	T01C3.5	*irld-14*	Q	K	common
V	16042823	G	C	T10C6.14	*his-1*	G	R	unique
V	17615382	C	T	Y6G8.5	Y6G8.5	E	K	unique
V	18270887	G	T	Y37H2C.4	Y37H2C.4	V	F	unique
V	19069825	G	A	Y39B6A.18	Y39B6A.18	P	L	unique
X	6101541	T	C	C15H9.4	C15H9.4	M	T	common
X	6759672	C	T	R03E9.3	*abts-4*	E	K	common
X	7304664	G	A	K03A1.5	*sur-5*	A	V	common
X	10036124	C	T	ZC374.2	ZC374.2	D	N	unique
X	16473719	C	T	F38E9.1	F38E9.1	P	L	unique

JK952 Chr.	Nucleotide position	Nucleotide change Ref.	JK952	ORF	Gene name	Ref A. A.	JK952 A. A.	Status
I	2587749	G	A	K07A3.2	*ptr-12*	R	*	unique
I	7092902	C	T	F22D6.5	*prpf-4*	A	T	common
I	7353179	C	G	F07A5.2	F07A5.2	P	A	common
I	7624508	G	A	F29D11.1	*lrp-1*	V	M	unique
I	8651317	G	A	F39H2.2	*sig-7*	R	K	common
I	8838166	C	T	F36A2.13	F36A2.13	A	T	distinct mutation in JK946
I	10807773	G	A	C35E7.9	C35E7.9	D	N	unique
I	12115770	C	A	M01G12.7	M01G12.7	L	F	unique
I	12358969	G	A	T09E11.5	*oac-44*	A	T	unique

I	13484417	A	G	Y48G10A.2	Y48G10A.2	I	T	common
I	14334390	G	A	F49B2.6	F49B2.6	V	I	common
I	15053241	A	G	F31C3.5	*psf-2*	K	E	common
II	3652474	G	A	F19B10.11	F19B10.11	A	T	unique
II	4870099	G	A	F11G11.11	*col-20*	P	L	unique
II	12152415	T	G	Y57A10A.4	Y57A10A.4	D	E	common
II	12633145	G	A	Y38E10A.17	Y38E10A.17	A	V	unique
II	15026573	G	A	Y53F4B.13	Y53F4B.13	V	I	common
III	15852	G	T	H10E21.1	H10E21.1	D	Y	common
III	1103278	G	A	F58B6.2	*inft-1*	G	S	unique
III	1843010	G	A	Y39A3CL.1	Y39A3CL.1	W	*	unique
III	2309106	A	G	Y54F10BM.20	Y54F10BM.20	D	G	common
III	5195074	C	A	K10D2.1	K10D2.1	T	K	common
III	5762753	G	A	B0244.2	*ida-1*	D	N	unique
III	5864877	T	C	F01F1.4	*rabn-5*	L	S	common
III	7197267	G	A	F37C12.13	*exos-9*	P	S	unique
III	7325159	G	T	F56C9.11	F56C9.11	P	H	common
III	8171519	C	T	C50C3.6	*prp-8*	P	L	unique
III	8440137	C	T	ZK1236.7	ZK1236.7	G	E	common
III	8888935	C	T	ZK637.14	*unc-32*	M	I	*e189*
III	8975983	C	T	R08D7.6	*pde-2*	G	E	common
III	9098563	G	A	F02A9.6	*glp-1*	G	E	*q231*
III	9201577	G	A	ZC84.1	ZC84.1	P	L	common
III	10111388	C	T	T16H12.9	T16H12.9	P	L	common
III	12219151	C	A	Y75B8A.16	Y75B8A.16	T	K	common
III	12474007	G	A	Y49E10.23	*cccp-1*	E	K	unique

III	12475401	G	A	Y49E10.23	*cccp-1*	A	T	common
III	13398942	C	T	F11F1.1	F11F1.1	A	V	common
IV	752771	G	A	Y55F3C.3	*kvs-5*	G	E	unique
IV	7994439	T	G	Y42H9B.2	*rig-4*	E	A	common
IV	12375120	T	A	F28D1.2	F28D1.2	T	S	unique
IV	13444300	G	A	K09B11.10	*mam-3*	T	I	unique
IV	13486920	C	T	Y45F10A.6	*tbc-9*	E	K	unique
IV	14238973	G	A	Y64G10A.1	Y64G10A.1	T	I	unique
V	1096463	G	A	Y50D4B.3	Y50D4B.3	E	K	unique
V	4791394	G	A	R02F11.10	R02F11.10	D	N	unique
V	4982300	T	C	C13D9.6	*srsx-13*	D	G	common
V	5496415	C	T	T05C3.2	T05C3.2	S	F	unique
V	7142145	A	C	F10D2.6	*ugt-37*	L	R	common
V	7220103	A	T	C13F10.6	C13F10.6	L	H	common
V	7508048	C	T	C54F6.10	*str-31*	G	E	unique
V	7892362	C	A	F40A3.6	F40A3.6	R	I	common
V	8760428	T	C	B0507.10	B0507.10	K	E	common
V	9749988	G	A	F58E6.4	F58E6.4	L	F	unique
V	12042395	G	A	F10C2.4	F10C2.4	V	I	unique
V	12047573	G	A	F10C2.5	F10C2.5	V	I	unique
V	14286334	G	A	F40G12.11	F40G12.11	M	I	unique
V	14786180	C	T	C48G7.3	*rin-1, tag-333*	M	I	common
V	14997197	C	A	T01C3.5	*irld-14*	Q	K	common
V	17498432	C	T	K03D7.4	*srh-261*	L	F	unique
V	17844064	C	T	Y59A8A.4	*srh-286*	S	N	unique
V	20816400	C	T	T03D8.7	T03D8.7	A	V	unique

X	695671	C	G	F02G3.1	*ncam-1*	Q	E	unique
X	951438	G	A	F53H8.2	*arr-1*	D	N	unique
X	5606168	C	T	F14D12.1	F14D12.1	P	L	unique
X	6005556	C	T	F22F4.1	F22F4.1	Q	*	unique
X	6006001	C	T	F22F4.1	F22F4.1	Q	*	unique
X	6101541	T	C	C15H9.4	C15H9.4	M	T	common
X	6476046	C	T	C15B12.8	C15B12.8	P	L	unique
X	6759672	C	T	R03E9.3	*abts-4*	E	K	common
X	7304664	G	A	K03A1.5	*sur-5*	A	V	common
X	10275758	G	A	F41E7.2	F41E7.2	R	H	unique
X	12725682	C	T	F16H9.1	*rgs-2*	P	S	unique
X	17229918	C	T	T25G12.6	T25G12.6	D	N	unique

"Common" status indicates a mutation that is present in both *sog-1* strains; as expected, these include *glp-1(q231ts)* and the linked marker mutation, *unc-32(e189)*. "Unique" status indicates a gene that is mutated only in JK946 or JK952. F36A2.13 is the only ORF to contain a distinct mutation in each strain. A. A., amino acid; Ref., reference genome; Chr., chromosome. Nucleotide coordinates refer to genome version WS240.
