# Supplementary material for: UBR-5, a Conserved HECT-Type E3 Ubiquitin Ligase, Negatively Regulates Notch-Type Signaling in Caenorhabditis elegans
Source: G3 (Bethesda). 2016 May 13;6(7):2125–34. doi: 10.1534/g3.116.027805 (PMC4938665; doi:10.1534/g3.116.027805)
Supplement: Supplemental Material [file supp_g3.116.027805_TableS2.pdf]

Table S2 Sequence changes associated with *ubr-5* alleles

Allele	Nucleotide change [a]	Amino acid change
q308	G554→A	A124→T
q345	C3793→T	R1026→stop
om2	4918-5022 deleted; C7492→T [b]	Deletion shifts the ORF, inserting 45 aa downstream of residue 1354; UBR-5 sequence deleted from residue 1355 to C-terminus
q298	G7325→A	G2442→D
q295	G9328→A	G2672→E
q303	G9771→A	G2803→E
q305	G10109→A	G2881→E
ok1108	9206-10565 deleted & 73 nt inserted	9 aa insertion downstream of residue 2631; UBR-5 sequence is deleted from residue 2631 to C-terminus

ORF, open reading frame; nt, nucleotide; aa, amino acid. Mutations are listed in order from 5' to 3' end of the gene.

[a] Number refers to nucleotide position in the gene sequence as listed in wormbase.org. The coding sequence (start codon) begins at nucleotide position 127.

[b] The single nucleotide substitution in *om2* is located C-terminal to the premature stop codon generated by the upstream deletion and therefore is not predicted to change an amino acid.
